# Gene Expression Patterns in Myelodyplasia Underline the Role of Apoptosis and Differentiation in Disease Initiation and Progression

**Published:** 2008-05-29

**Authors:** Merav Bar, Derek Stirewalt, Era Pogosova-Agadjanyan, Vitas Wagner, Ted Gooley, Nissa Abbasi, Ravi Bhatia, H. Joachim Deeg, Jerald Radich

**Affiliations:** 1 Clinical Research Division, Fred Hutchinson Cancer Research Center, Seattle, Washington; 2 City of Hope National Medical Center, Duarte, California

**Keywords:** myelodysplasia, gene expression, apoptosis, differentiation

## Abstract

The myelodysplastic syndromes (MDS) are clonal stem cell disorders, characterized by ineffective and dysplastic hematopoiesis. The genetic and epigenetic pathways that determine disease stage and progression are largely unknown. In the current study we used gene expression microarray methodology to examine the gene expression differences between normal hematopoietic cells and hematopoietic cells from patients with MDS at different disease stages, using both unselected and CD34+ selected cells. Significant differences between normal and MDS hematopoietic cells were observed for several genes and pathways. Several genes promoting or opposing apoptosis were dysregulated in MDS cases, most notably MCL1 and EPOR. Progression from RA to RAEB(T) was associated with increased expression of several histone genes. In addition, the RAR-RXR pathway, critical for maintaining a balance between self-renewal and differentiation of hematopoietic stem cells, was found to be deregulated in hematopoietic cells from patients with advanced MDS compared to patients with refractory anemia. These findings provide new insights into the understanding of the pathophysiology and progression of MDS, and may guide to new targets for therapy. Taken together with previous published data, the present results also underscore the considerable complexity of the regulation of gene expression in MDS.

## Introduction

The myelodysplastic syndromes (MDS) are a heterogeneous group of clonal disorders of the hematopoietic stem cells [1, 2]. The natural history of MDS is that of progressive cytopenia with increasing transfusion needs, infectious and bleeding complications or alternatively, evolution to secondary AML [1, 2]. While relatively simple clinical and pathologic scoring systems with prognostic relevance have been developed, the molecular mechanisms involved in evolution of the disease are largely unknown [3, 4]. Identifying molecular markers of MDS may allow for a more accurate assessment of the prognosis and potentially identify new targets for therapy.

The genetic lesions so far identified in MDS incompletely describe the biology and heterogeneity of the disease. Clonal karyotypic abnormalities are observed in approximately 40–50% of patients with primary MDS, and 90% of therapy-related MDS [2, 5]. Mutations important in denovo AML, for example mutations in RAS protooncogenes and FLT3 internal tandem duplications (ITD), have been described in 5%–20% of MDS patients and are variably associated with disease progression [3, 6–9]. Beside overt genetic lesions, epigenetic lesions may also play a roll in the development of MDS. Hypermethylation has been described in many malignancies including MDS and may be associated with disease development, progression and prognosis [10–14]. For example, p15 promoter hypermethylation has been shown to be associated with MDS progression to AML in some studies [3, 15]. However, none of these alterations are specific for MDS and the underlying molecular causes of MDS have remained poorly understood.

The vast number of genetic and epigenetic disturbances in MDS makes investigations to identify potential common pathways that may involved in disease development and progression challenging. Oligonucleotide microarrays have been found to be an excellent tool to study biology and identify potential prognostic factors in many forms of malignancies, including MDS. This platform allows examination of thousands of genes using a single sample. In the current study we used oligonucleotide microarrays to determine how the genetic expression profile differs between healthy hematopoietic cells and hematopoietic cells from patients with MDS, and to identify genes and pathways that might be relevant for MDS evolution and progression.

## Materials and Methods

### Patients

Mononuclear cells (N = 35) or purified CD34+ cells (N = 8) from the marrow of 43 MDS patients were studied. Patients were sub-grouped according to the French-American-British (FAB) classification [16] into refractory anemia (RA, N = 18), refractory anemia with ringed sideroblasts (RARS, N = 11), refractory anemia with excess blasts (RAEB, N = 8), and refractory anemia with excess blasts in transformation (RAEB-t, N = 1). In addition, we included one patient with unclassified MDS, one patient with a mixed MDS/myeloproliferative picture, and three patients with AML that had evolved from MDS. Three patients had 5q deletion, however not as an isolated lesion, but as part of complex cytogenetic abnormalities. Mono-nuclear (N = 10) or purified CD34+ bone marrow cells (N = 14) from 24 healthy subjects were used as controls. Samples from five normal bone marrows, four patients with low grade MDS (RARS or RA), and seven patents with high grade MDS (RAEB-1 or RAEB-2) not used in the microarray studies were used for PCR validation studies of MCL1 expression. Some of the patients had received treatment in the past, including chemotherapy, erythropoietin or thalidomide, but no treatment was given within 4 weeks of sample acquisition. All patients and healthy donors had given informed consent according to the requirements of the Institutional Review Board.

### Sample preparation

Heparinized bone marrow samples were obtained by aspiration from the posterior iliac crest. Mono-nuclear cells were separated by density gradient centrifugation through Ficoll-Hypaque. CD34+ cells were purified by two rounds of high-gradient magnetic cell separation using autoMACS (Milt-enyiBiotecInc, Auburn, CA)with superparamagnetic microbead labeling of CD34+ cells. Total RNA was extracted using TRIzol (Invitrogen, Carlsbad, CA.) according to the manufacturer’s protocol. All RNA samples were analyzed on an HP 2100 bio-analizer (Aglient Technologyies, Palo Alto, CA USA) to ensure the integrity of total RNA prior to use in microarray assays [17].

### Oligonucleotide microarray gene expression

RNA obtained from mononuclear cells was prepared according to the standard Affymetrix protocol (GeneChip Expression Analyses Technical Manual (http;//www.affymetrix.com/support/technical/manual/expression_manual.affx). For CD34+ cells, RNA was prepared using a single stranded linear amplification protocol (SLAP) prior to RNA labeling and hybridization [17]. Fragmented, bio-tinylated cDNA was hybridized to an Affymetrix HG-U133 microarray according to the manufacturer’s protocol.

### Data analysis

DAT files for individual samples were generated using Affymetrix MAS 5.0 software. Target signals for probe sets were scaled to 500 for analyses. The detection algorithm was based upon default settings per Affymetrix recommendations (https://www.affymetrix.com/support/downloads/manuals/data_analysis_fundamentals_manual.pdf).

Signals were transformed into log_2_ intensity. Normalization was performed using the R 2.2.1 software [18]. The expression data was then analyzed using SAM 2.2.1 (Significance Analysis of Microarrays (SAM), Stanford, CA)[19] or Gene-Plus 1.2 (http://www.enodar.com/technology6.htm) for specific gene expression analysis and Gene Set Enrichment Analysis software (GSEA v1.0, Broad Institute, MIT, Cambridge, MA) for pathway analysis [20]. We selected a false discovery rate (FDR) of 5% to determine statistically significant up or down regulated genes in SAM [21]. Hierarchical clustering was performed using the dChip software available at http://biosun1.harvard.edu/complab/dchip/.

For the analysis of MCL1 levels and transcript ratio in different MDS disease stages, a global p-value was derived using linear regression and tested in the null hypothesis that the mean transcript ratios were the same across normal, RA, and advanced phase MDS. The significant test for trend used linear regression of the mean of the transcript across each group, where each group was assigned values 1, 2, and 3 for normal, low, and advanced MDS, respectively.

### PCR validation of MCL1

To validate the gene expression of MCL1 in normal and MDS hematopoesis, we developed quantitative RT-PCR assays. Samples from five normal bone marrows, four patients with low grade MDS (RARS or RA), and seven patents with high grade MDS (RAEB-1 or RAEB-2) not used in the micro-array studies were analyzed. For MCL1, the two alternative splice variants were amplified separately, producing a full-length transcript (T×1) associated with anti-apoptosis, and the alternatively spliced, smaller transcript (T×2) that is pro-apoptotic. Quantitative PCR validation for MCL1 splice variants was performed on an ABI 7900 HT Fast Real-Time PCR System. Thermocycler conditions were set at: 50 °C for 2 min, 95 °C for 10 min, and 40 cycles at 95 °C for 15 sec and 60 °C for 1 min. A common primer sequence was used for the forward MCL1 primer (E×1): 5′-GAAGGCGCTGGAGACCTTAC-3′). MCL1-T×1 (MCL1-T×1R) and MCL1-T×2 (MCL1-T×1R) reverse primer sequences were 5′-TTTCCGAAGCATGCCTTGG-3′ and 5′-ACTCCACAAACCCATCCTTGG-3′, respectively. All probes were FAM-TAMRA; the MCL-1 probe sequence was 5′-ATGGCGTGCAGCG-CAACCAC-3′. Controls genes were obtained from cloned 2.1 TOPO vector plasmids. The size of the MCL1-T×1 and MCL1-T×2 inserts was 517 bp, and 269 bp respectively.

## Results

To explore the changes in gene expression that occur with the evolution of MDS from normal hematopoietic progenitor cells and during disease progression from RA to RAEB and transformation into secondary AML, we used two general strategies. First, we identified expression changes common to *all* MDS cases compared to normal bone marrow, and secondly, we examined gene expression in MDS cases that correlated with disease subtypes. Since it is not clear to what extent the biology of MDS is determined only by the malignant “stem cell” and what the entire cellular environment contributes, separate analyses were performed in unselected and CD34+ selected populations of both MDS and normal hematopoietic cells.

### Gene expression in MDS compared to normal bone marrow

**Figure 1:**
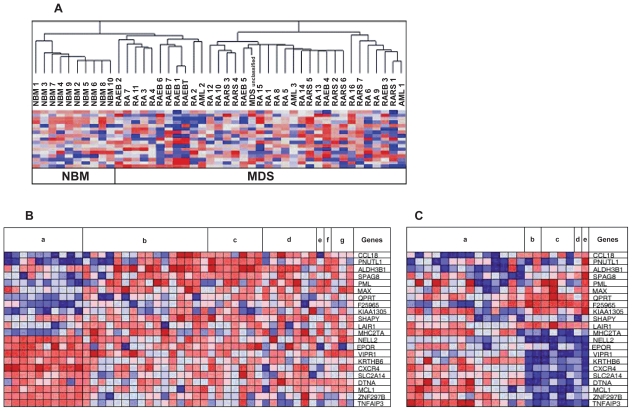
**A. Hierarchical clustering of 1,000 differentially expressed genes in normal marrow and marrow from MDS patients.** Each row represents a single probe set and each column a separated normal or MDS marrow sample. Blue coloring represents down-regulated genes in MDS compared to normal, while red represents up-regulated genes in MDS. Unsupervised hierarchical clustering showed complete segregation between normal bone marrow (n = 10) and MDS (n = 35), but no segregation between the different stages of MDS. **B, C. Heat map of up- and down-regulated genes in unselected mononuclear and CD34+ selected cells from MDS marrow and normal marrow samples.** B) Comparison of gene expression in unselected mononuclear cells in MDS and normal bone marrow: “a”—normal bone marrow samples; “b”—RA samples; “c”—RARS; “d”—RAEB; “e”—RAEB-t; “f”—unclassified MDS; and “g”—AML cases arising from antecedent MDS. C) Comparison of gene expression in CD34+ selected cells: “a”—normal marrow; “b”—RA; “c”—RARS; “d”—RAEB; and “e”—a case of MDS/MPS (a mixed MDS/myeloproliferative picture).

We first compared unselected marrow mononuclear cells from 35 patients with MDS and 10 healthy donors. Unsupervised hierarchical clustering showed complete segregation between normal bone marrow and MDS marrow (Fig. 1A). However, there was no segregation between the different morphologic subtypes of MDS (Fig. 1A), underscoring the difficulty of describing MDS biology by morphology alone.

In comparing all MDS cases to normal bone marrow, 516 genes were up-regulated and 2107 were down-regulated in the MDS samples (Supplementary Table 1). The top up-regulated genes in MDS included: the HSPA1A (heat shock 70 kDa protein), CEACAM6 (carcinoembryonic antigen-related cell adhesion), DEFA1/4 (defensin alpha 1 and 4), GFI1 (growth factor independent 1) and TCN1 (transcobalamin 1). Among the top down-regulated genes were CREM (a cAMP responsive element modulator), SC5D (sterol-C5-desaturase (ERG3 delta-5-desaturase homolog, fungal)-like), PIK3R1 (phosphoinositide-3-kinase, regulatory subunit 1 (p85 alpha) and IRF4 (interferon regulatory factor 4). GSEA analysis was then used to discover annotated biological pathways over-represented by genes that were up or down-regulated in MDS compared to normal bone marrow. The pathways most significantly enriched in abnormally regulated genes in MDS were vesicle transport, glycogen metabolism, chaperone modulated interferon signaling, and the pentose phosphate pathway.

**Figure 2:**
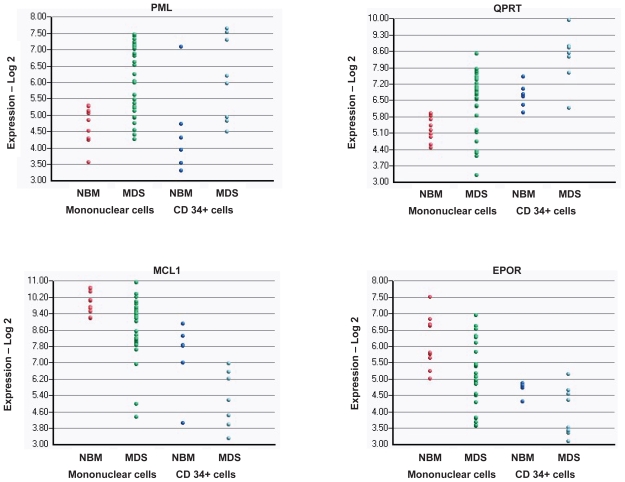
Expression of genes that were up- or down-regulated in MDS compared to normal bone marrow Log-2 expression level is shown on the Y axis; samples and cell type are indicated on the X axis. For each one of the four genes shown in this figure, the expression of the individual samples (for both unselected mononuclear cells and CD34+ selected cells) are shown. NBM: normal bone marrow.

We next compared gene expression in CD34+ selected cells from MDS and normal bone marrow to examine potential changes occurring primarily in the putative MDS “stem cell”. We identified 704 genes that were up-regulated and 826 genes that were down-regulated in the CD34+ cells from MDS patients compared to CD34+ cells from healthy donors (Supplementary Table 2). In comparing the genes that differed significantly between unsorted samples and purified CD34+ cells, we observed an overlap of 12 genes that were consistently up-regulated in *both* the unselected and CD34+ selected cells, and 95 genes that were consistently down-regulated in *both* the unselected and CD34+ selected cells (Table 1, full list in Supplementary Table 3). These genes are likely to be highly relevant as markers of biological activity, as well as targets for diagnostic and therapeutic tools. The top overlap genes and their expression in all samples are shown in Figure 1B, 1C and Figure 2. The overlap genes fall into several relevant biological categories. Interestingly, many genes were deregulated in favor of increased apoptosis: decreased expression in the anti-apoptotic regulator MCL1, the erythropoetin receptor EPOR, and TNF anti-apoptotic modulator TNFAIP3, and an increased expression in Ca+2 activated nucleotidase CANT1, and the inhibitory receptor LAIR1. There was no association of EPOR level and past use of erythropoetin. Dysregulated immune function and cytokine expression have been implicated increased expression of the CD4/CD8 cytokine CCL18, the decreased expression of the “master” control gene for class II MHC expression CIITA, and the decreased expression of CXCR4, the receptor for stroma derived factor 1.

### Gene expression in RA compared to advanced MDS

**Figure 3:**
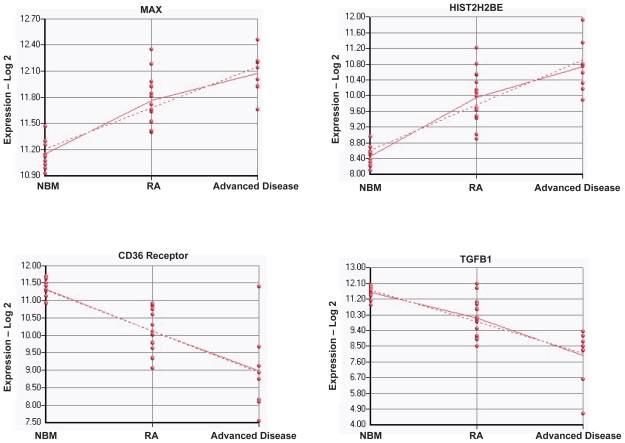
Expression of genes that correlated with advanced disease Log-2 gene expression of MAX, HIST2H2BE, CD36 Receptor and TGFB1 in normal bone marrow (NBM), RA, and advanced disease (RAEB, RAEB-t, AML).

The signals that lead to progression from RA to more advanced disease are poorly understood. We compared unselected mononuclear cells from 16 patients with RA to unselected mononuclear cells from 11 patients with advanced MDS, again using normal bone marrow as reference. Several genes were expressed differently in normal bone marrow, RA and advanced disease (Table 2, Supplementary Table 4). Among these genes are MAX (MYC associated factor X), HIST2H2BE (histone 2, H2be), HIST2H2AA (histone 2, H2aa), HIST1H2BG (histone 1, H2bg) and TNFRSF1A (tumor necrosis factor receptor superfamily, member 1A) which were increasingly expressed with the evolution of RA from normal bone marrow and with progression from RA to more advanced stages of MDS (Fig. 3). ASGR2 (asialoglycoprotein receptor 2), TGFB1 (transforming growth factor, beta 1) IDH3B (isocitrate dehydrogenase 3 NAD+ beta) and EPB41L3 (erythrocyte membrane protein band 4.1-like 3) were down regulated in RA compared to normal bone marrow, and were further down-regulated in advanced MDS (Fig. 3). We next searched for changes in biological pathways associated with MDS disease progression. Using the GSEA software we identified statistically significant enrichment in genes involved in the Rac 1 cell motility signaling pathway and the RAR-RXR pathway with advanced disease.

### Validation studies of MCL1

**Figure 4:**
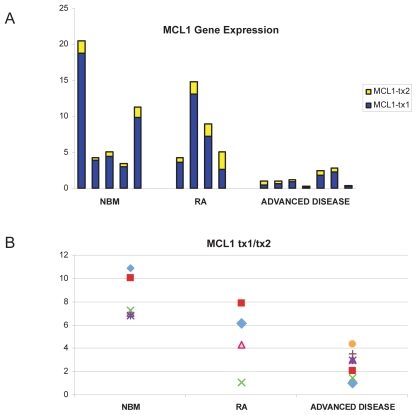
Quantitative RT-PCR assay of MCL1 **A. Expression of MCL1 anti-apoptotic transcript (t×1) and pro-apoptotic transcript (t×2).** Values on the y axis represent MCL1 expression relative to endogenous control (beta-2-microglobulin) in normal bone marrow (n = 5), RA patients (n = 4) and MDS patients with advanced disease (n = 7). Both normal and MDS marrows were obtained from individuals who were not used for the gene expression array. **B. Relative expression of MCL1 anti-apoptotic transcript (t×1) versus pro-apoptotic transcript (t×2).** Values on the y axis represent the relative expression of MCL1 t×1 versus t×2 for each of the individuals shown in panel A.

In validation studies (Fig. 4), the expression of both the longer anti-apoptotic transcript (T×1) and the shorter pro-apoptotic transcript (T×2) variants of MCL1 decreased significantly from normal bone marrow to low grade, and high grade MDS (global significance levels of T×1 and T×2 when comparing normal, low, and high grade MDS were p = 0.03 and p = 0.007, respectively). Moreover, there was a shift of the ratio of the anti-apoptotic/pro-apoptotic mRNA level in these three states, with a T×1/T×2 of 8.4 for normal bone marrow, 4.8 for low grade MDS, and 2.6 for high grade MDS (global significance p = 0.0008, test of trend p = 0.0001). Thus, MDS and progression of MDS were associated with not only a global decline in MCL1 level, but a shift in transcript towards a pro-apoptotic bias.

## Discussion

MDS comprises a heterogeneous group of clonal disorders that are characterized by aberrant differentiation in multiple hematopoietic cell lineages and are thought to involve hematopoietic stem cells [1, 2]. However, there is mounting evidence that the disease process is not entirely stem cell-autonomous and that signals derived from more differentiated cells, in particular monocytes and T lymphocytes, and from the marrow stroma affect the disease process [22–30]. We, therefore, performed an analysis of gene expression in both unselected mononuclear cells and in selected CD34+ cells from MDS marrow in comparison to the analogous cell populations from normal marrow. Our results identified 2623 genes with expression differences between unselected marrow mononuclear cells from healthy donors and MDS patients and 1530 genes with expression differences between CD34+ cells from healthy donors and MDS patients. Compared to normal marrow, MDS was associated with an aberrant expression of genes involved in apoptosis, including a decreased expression of MCL1 and EPOR1, and these changes were present both in non-selected and CD34+ selected cell populations. Moreover, the PML gene and genes of the RAR-RXR pathway were found to be associated with the diagnosis of MDS and with advanced disease, respectively, suggesting disruptions of the normal differentiation pathway.

Several genes associated with the promotion of a pro-apoptotic state were identified, including anti-apoptotic regulator MCL1, the erythropoetin receptor EPOR, and TNF anti-apoptotic modulator TNFAIP3. Down regulation of MCL1 is consistent with the increased rate of apoptosis observed in MDS [26, 31–35]. The protein encoded by the MCL1 gene belongs to the Bcl-2 family, known to be regulator of programmed cell death. MCL1 has been shown to be essential in the survival of hematopoetic stem cells, as inducible deletions of MCL1 in murine models results in a profound loss of bone marrow function, including a loss of hematopoietic stem cells [36]. MCL1 activity appears to be required for neutrophil, but not for macrophage survival, [37] suggesting the possibility of lineage specific or differentiation dependent activity. Alternative splicing of the MCL1 gene results in two transcript variants encoding distinct isoforms. The longer gene product (isoform 1; T×1) enhances cell survival by inhibiting apoptosis, while the alternatively spliced shorter gene product (isoform 2; T×2) promotes apoptosis and is death-inducing [38]. These findings are reminiscent of those described for the long and short splice variants of the death signal inhibitory protein FLIP in MDS, [39] suggesting that regulation of splice variants at the transcriptional level is involved in the determination of cell death in MDS. Our data show that not only was MCL1 expression decreased in MDS compared to normal hematopoetic cells, but ratio of long/short transcripts shifted in favor of a more pro-apoptotic state. Would such a pattern be compatible with the general observation that apoptosis in marrow cells overall tends to decrease as MDS progressed to more advanced stages? [40] The results appear counterintuitive. However, we have previously shown that the rate of apoptosis differs between clonal and non-clonal hematopoietic cells, and the relative proportions of those cell populations change with progression of MDS [41]. Further we observed that expression of the short splice variant of FLIP, characterized anti-apoptotic protein, showed a positive correlation with the extent of apoptosis [39]. Taken together with the lineage specificity of MCL1 as described by Dzhagalove et al. [37] it is conceivable that a pro-apoptotic effect of MCL1 in advanced MDS is expressed only in subset of cells, but does not interfere with increasing proliferation of the malignant clone. Such a model would also be consistent with the observed overall decline in expression of this gene as MDS progresses (see results).

The erythropoietin receptor (EPOR) is a member of the cytokine receptor family. Upon erythropoietin binding, the erythropoietin receptor activates the Jak2 tyrosine kinase, which in turn activates various intracellular signaling pathways, including, Ras/MAP kinase, phosphatidylinositol 3-kinase and STAT transcription factors [42]. EPOR has an anti-apoptotic function via the Akt-pathway, and signaling via the erythropoietin receptor promotes erythroid cell survival, particularly in patients with MDS [43–45]. Thus, the down-regulation of both MCL1 and EPOR may play a role in the dysregulation of apoptosis in hematopoetic cells leading to ineffective hematopoiesis in MDS. It is clear, however, that the pattern of expression of MCL1 and EPOR by themselves can not explain satisfactorily the extent of apoptosis and proliferation dysregulations at different stages of MDS. Other factors are involved, and studies of purified cell populations, simultaneously analyzing the impact of various signals will be necessary [46].

Of special biological interest are the MAX (MYC associated factor X) and PML (promyelocytic leukemia) genes, which were found to be up-regulated in both unselected mononuclear cells and CD34+ selected cells in MDS compared to normal bone marrow and which showed a correlation with progression to advanced disease. The protein encoded by the MAX gene is a transcription factor that interacts with the MYC oncoprotein to form homodimers and heterodimers. Rearrangement among these dimer forms provides a complex system of transcriptional regulation [47]. Therefore, alteration in transcription regulation resulted by up regulation of the MAX gene might play a role in the pathophysiology of MDS. In addition, the correlation with progression to advanced disease and the fact that the MAX gene was found to be up regulated in both CD34+ selected cells and unselected marrow cells make this gene a candidate marker for disease progression. The protein encoded by the PML gene is a member of the tripartite motif (TRIM) family. This phosphoprotein localizes to nuclear bodies where it functions not only as a transcription factor but also as a tumor suppressor. Its expression is cell-cycle related and regulates the p53 response to oncogenic signals [48]. The gene is also involved in the translocation of the retinoic acid receptor alpha gene associated with acute promyelocytic leukemia (APL) [49]. A Further suggestion of the importance of PML and the RARA pathway in MDS disease progression is the deregulation of the RAR-RXR pathway in the advanced MDS noted in our analysis. RXR and RAR are nuclear receptors that bind either all-trans retinoic, 9-cis retinoic acid, or other retinoid ligands [50]. Ligand binding induces a conformational change in the receptors which results in dissociation of the co-repressors and binding of co-activators with histone acetylase activity [50]. The retinoic acid pathway is critical for maintaining a balance between self-renewal and differentiation of hematopoetic stem cells, [51, 52] and deregulation of the RAR-RXR pathway, as was shown in our study, may affect differentiation and self-renewal, and thereby allow RA to progress to acute leukemia.

Three histone genes (HIST2H2BE, HIST-2H2AA, HIST1H2BG) were found to be up-regulated in MDS compared to normal bone marrow, and moreover found to be correlated with advanced disease. The expression of specific histone classes dictate changes in chromatin structure and gene expression, and influences various pathways, including cell cycle progression [53–55]. In addition, there is considerable evidence that histone H1 also functions as a non-specific repressor of transcription [56]. Moreover, post-translational modifications of histones by histone acetylation and deacetylation play a role in tumorgenesis. Histone deacetylases are promising targets in drug development for cancer therapy [57]. Given that our data suggest that aberrations in histone biology are involved in MDS progression, their may be a rationale for using these agents to stem progression of early MDS, especially in combination with agents that block apoptosis, as discussed above.

Several studies have examined gene expression in MDS, using either purified CD34+ or unselected mononuclear cells [58–62]. There is little overlap between the genes identified across those studies or our current results. Such discrepancies are not uncommon in gene expression studies, and likely result from differences in cell types studied (CD34+ versus mononuclear cells), analytic techniques to define significant genes, and composition of the cohorts of patients studied. For example, the study by Pellagatti et al. [60] examined CD34+ cells from 55 MDS patients obtained from multiple centers, and showed that MDS cells had gene signatures enriched in interferon response genes. Of the 55 patents, 20 had the 5q- chromosomal aberration. Patients with 5q- MDS are unusually sensitive to the drug lenalidomide, and have recently been shown to have a unique gene expression signature using unselected mononuclear cells [63].

Since it is unknown which cell population, CD34+ selected or unselected mononuclear cells, best describes the pathology of MDS, and given that various studies have used both types of samples, we performed both types of arrays, and focused on genes found to be dysregulated in both cell populations. Thus, we consider our gene selection to be fairly robust. We are comforted by the fact that the most relevant genes identified in the present analysis seem biologically relevant: alterations of differentiation and proliferation pathways (PML, RAR-RXR, MAX), involvement in apoptosis (MCL1, EPOR) and regulation of hematopoiesis (TNFAIP3, CXCR4, CCL18).

Since the patient population included in this study was relatively small, we were unable to study specific correlations between gene expression, cytogenetics, and clinical presentation. However there did not appear to be an association of previous treatment to the molecular signature, and specifically there was no effect of erythropoietin treatment on EPOR gene expression. These findings strengthen our interpretation that low EPOR levels might be related to the pathophysiology of MDS, and might explain the heterogeneity of response to treatment with erythropoietin among MDS patients.

In conclusion, this study provides new data on gene expression in the different phases of MDS. Although no single gene can likely explain the pathophysiology of the disease, some of the differences delineated in this study may prove relevant in our efforts to identify prognostic markers and therapeutic targets.

## Supplementary Tables

Table S1 MDS (multiple groups) vs NBM mononuclear cells.

Table S2 MDS (multiple groups) vs NBM CD34+ cells.

Table S3 OVELAPPED GENES (up/down regulated) in MDS multiple groups vs NBM (Mononuclear cells and CD 34+ cells).

Table S4 Advanced Disease (RAEB and RAEBT) versus RA versus normal bone marrow.

## Figures and Tables

**Table 1:** Top genes differentially expressed in MDS compared to normal bone marrow.

Gene symbole	Description	CD34+ cells:			Mononuclear cells:		
		SAM score^1^	Fold change	q-value(%)^2^	SAM score	Fold change	q-value(%)
**Up regulated genes:**							
MAX	MAX protein	5.56	1.67	0.00	2.84	3.28	2.61
PML	promyelocytic leukemia	4.65	2.67	0.21	3.61	4.39	1.52
PNUTL1	septin 5	3.57	4.22	1.63	3.94	3.35	0.75
SHAPY	calcium activated nucleotidase	3.18	5.59	1.95	4.46	3.05	0.21
MHC2TA	MHC class II transactivator	2.90	4.03	2.21	3.77	3.09	1.14
ALDH3B1	aldehyde dehydrogenase 3 family, member B1	2.67	2.15	3.03	4.63	1.85	0.21
CCL18	chemokine (C-C motif) ligand 18 (pulmonary and activation-regulated)	2.62	12.50	3.03	3.87	5.61	0.94
SPAG8	sperm associated antigen 8	2.53	2.66	3.37	4.41	2.00	0.21
QPRT	quinolinate phosphoribosyltransferase (nicotinate-nucleotide pyrophosphorylase (carboxylating))	2.46	3.59	3.37	3.32	3.01	3.11
LAIR1	leukocyte-associated Ig-like receptor 1	2.21	1.84	4.76	4.16	1.63	0.34
**Down regulated genes:**							
VIPR1	vasoactive intestinal peptide receptor 1	−4.97	0.18	0.00	−2.66	0.69	3.11
SLC2A14	solute carrier family 2 (facilitated glucose transporter), member 14	−4.12	0.27	0.00	−2.71	0.65	3.11
MCL1	myeloid cell leukemia sequence 1 (BCL2-related)	−4.03	0.21	0.00	−3.32	0.61	1.14
TNFAIP3	tumor necrosis factor, alpha-induced protein 3	−3.78	0.18	1.31	−4.50	0.44	0.32
DTNA	dystrobrevin, alpha	−3.77	0.24	1.31	−2.47	0.78	4.63
NELL2	NEL-like 2 (chicken)///NEL-like 2 (chicken)	−3.72	0.33	1.31	−3.17	0.63	1.43
EPOR	erythropoietin receptor	−3.71	0.35	1.31	−3.19	0.67	1.43
KRTHB6	keratin, hair, basic, 6 (monilethrix)	−3.66	0.37	1.31	−3.40	0.79	0.94
CXCR4	chemokine (C-X-C motif) receptor 4	−3.61	0.31	1.31	−3.80	0.56	0.56
ZNF297B	zinc finger protein 297B	−3.56	0.31	1.31	−3.81	0.50	0.56

1 Score—The *T*—statistic Value.

2 q-value—The lowest false discovery rate at which the gene is called significant (Like the “p value” adapted to analysis of a large number of genes).

**Table 2:** Top genes differentially expressed in Advanced Disease (RAEB and RAEBT) compared to RA compared to normal bone marrow.

Gene symbole	Gene description	Linear slope^1^	P (linear slope)
**Up regulated genes:**			
HIST2H2BE	histone 2, H2be	1.16	0.00
MAX	MAX protein	0.47	0.00
HIST2H2AA	histone 2, H2aa	1.52	0.00
TNFRSF1A	tumor necrosis factor receptor superfamily, member 1A	0.83	0.00
HIST1H2BG	histone 1, H2bg	1.08	0.00
ARHGEF2	rho/rac guanine nucleotide exchange factor (GEF) 2	0.37	0.00
SNX19	sorting nexin 19	0.29	0.00
C1RL	complement component 1, r subcomponent-like	0.87	0.00
H2BFS	H2B histone family, member S	1.37	0.01
HIST1H2BF	histone 1, H2bf	0.97	0.01
**Down regulated genes:**			
ASGR2	asialoglycoprotein receptor 2	−1.75	0.00
RNASE4	ribonuclease, RNase A family, 4	−1.11	0.00
IDH3B	isocitrate dehydrogenase 3 (NAD+) beta	−0.36	0.00
EPB41L3	erythrocyte membrane protein band 4.1-like 3	−1.70	0.00
TGFBI	transforming growth factor, beta-induced, 68 kDa	−1.80	0.00
CSPG2	Chondroitin sulfate proteoglycan 2 (versican)	−1.39	0.00
KIAA0399 (ZZEF1)	zinc finger, ZZ-type with EF-hand domain 1	−1.17	0.00
FLJ22222	hypothetical protein FLJ22222	−0.98	0.00
IL15	interleukin 15	−0.67	0.01
CLIC3	chloride intracellular channel 3	−1.86	0.01

1 Linear Slope—Change of value in the dependent variable (gene expression change) per unit of independent variable (disease stage); The changes in gene expression in the different stages of MDS.
